# *In silico* characterization of differentially expressed short-read nucleotide sequences identified in dieback stress-induced transcriptomic analysis reveals their role as antimicrobial peptides

**DOI:** 10.3389/fpls.2023.1168221

**Published:** 2023-03-20

**Authors:** Siddra Ijaz, Imran Ul Haq, Riffat Malik, Ghalia Nadeem, Hayssam M. Ali, Sukhwinder Kaur

**Affiliations:** ^1^ Centre of Agricultural Biochemistry and Biotechnology (CABB), University of Agriculture, Faisalabad, Pakistan; ^2^ Department of Plant Pathology, University of Agriculture, Faisalabad, Pakistan; ^3^ Department of Botany and Microbiology, College of Science, King Saud University, Riyadh, Saudi Arabia; ^4^ Department of Plant Pathology, University of California, Davis, Davis, CA, United States

**Keywords:** antimicrobial peptides, resistance genes, RGAs, homology modelling, fingerprints

## Abstract

We investigated the *in silico* characterization of short-length nucleotide sequences that were differentially expressed in dieback stress-induced transcriptomic analysis. They displayed homology with C-terminal flanking peptides and defensins-like proteins, revealing their antimicrobial activity. Their predicted fingerprints displayed protein signatures related to antimicrobial peptides. These short-length RGAs have been shown to possess structural motifs such as APLT P-type ATPase, casein kinase II (CK2), protein kinase 3, protein kinase C (PKC), and *N*-glycosylation site that are the attributes of disease resistance genes. The prediction of arginine and lysine residues in active binding sites in ligand docking analysis prophesied them as antimicrobial peptides due to their strong relation with antimicrobial activity. The *in silico* structural–functional characterization has predicted their role in resistance against microbial pathogens. Moreover, the predicted antimicrobial peptide regions showed their homology with the signature domain of PR-5-like protein and AMP family Thaumatin

## 1 Introduction

*Dalbergia sissoo*, widely known as sissoo, shisham, or tahli, is a well-known timber-producing tree indigenous to South Asian countries (Shah et al., 2010). It has multipurpose uses and finds applications in the forestry, agroforestry, and horticulture sectors. Its high-quality timber is utilized in various applications, e.g., furniture, building materials, and fabricated textiles. Dieback disease is a significant problem that leads to the destruction of this tree and financial loss. The development of this disease is attributable to several biotic and abiotic variables (Ahmad et al., 2015). Dieback was reported as an infectious disease in 1998 in Punjab province. Since then, the shisham decline has been considered an alarming situation in the subcontinent (Bajwa et al., 2003). Estimates showed that dieback had wreaked significant havoc on the planted trees, resulting in a disease incidence of 40% along roadsides and embankments in the Sindh province. Another survey in the Punjab province recorded a mortality rate of 20.5%–40.4% (Bajwa et al., 2003; Khan et al., 2004). The susceptibility of *D. sissoo* to pathogens leads to tree decline, and recurrent stress ultimately causes death. There is a dire need to preserve this valuable tree.

The use of resistant germplasm has been considered a viable approach to tackle this adverse issue, thus helpful in promoting disease-resistant breeding (Minocha and Jain, 2000). Plants are inert organisms with a constant risk of getting affected (Cramer et al., 2011). They possess several innate defense mechanisms that work as a barrier between plants and invading microbes, thereby enhancing plant productivity (Abdul Malik et al., 2020). Plants have a two-level immune system that protects them against infection. The primary level comprises pathogen-associated molecular patterns (PAMP)-triggered immunity in response to pathogen invasion. It operates when pattern recognition receptors (PRRs) on the host surface recognize PAMPs released by them upon invading plants (Meng and Zhang, 2013). This immunity results in physiochemical alterations in plants and thus indicates a particular microbial invasion. In a counterattack, phytopathogens release effector molecules that cause disease in the host. As a result, a secondary level of immunity called ETI responds to the effectors, resulting in programmed cell death (Tsuda and Katagiri, 2010). These pathogen-secreted effectors are identified particularly by R genes (Jones and Dangl, 2006).

The general class of *R* genes in plants encompasses NBS and LRRs and belongs to the STAND P-loop NTPase family, contributing to plant defense responses against pathogen attack (Meyers et al., 1999). The NBS-LRR proteins act as adaptable chaperones and are involved in plant resistance *via* signaling cascades, i.e., reactive oxygen species and tissue damage (McHale et al., 2006). Additionally, the expression analysis of NBS-LRR proteins has predicted the existence of motifs analogous to disease resistance genes, i.e., protein kinase 3, protein kinase C, the casein kinase II phosphorylation site, and the *N*-glycosylation site (Baldi et al., 2004).

Natural antimicrobial peptides (AMPs) typically comprise fewer than 60 amino acids with an l-configuration and molecular masses less than 10 kDa (Splith and Neundorf, 2011). The majority of AMPs are cationic and typically contain a significant amount of hydrophobic amino acids (≥ 30), and their net charges range from +2 to +9 (Hancock and Sahl, 2006). The amphipathic nature of AMPs is mainly because of the positively charged (lysine (Lys) and arginine (Arg) residues) and hydrophobic amino acids. This property allows AMPs to interact with the negatively charged membranes of the microbial cell, thus leading to bacterial death. Moreover, this is a core feature in the mode of action of the antimicrobial compounds (Arias et al., 2018; Semreen et al., 2018). AMPs are reported to have antifungal, antiparasitic, and antibacterial properties and have an essential role in the host’s innate immunity (Splith and Neundorf, 2011). They are significantly less likely to develop resistance regarding their mechanism of action against bacteria and have a faster killing activity (Zasloff, 2002). The small size of the AMPs enables them to be synthesized easily and can either kill or hinder the growth of microbes (Tincu and Taylor, 2004). The significant potential of AMPs permits them to serve as an alternative to next-generation antibiotics; however, clinical trials are still in progress (Fjell et al., 2012; Greber and Dawgul, 2017). The major limitations in the AMPs’ potential to be used in drug development are the absence of proteolytic stability, the high cost of production, and the unfavorable toxicity during drug administration (Fjell et al., 2012).

Plant pathogens are detrimental to the viability and survival of *D. sissoo* and can cause crucial economic losses. Recognizing resistance genes in plants is significant in developing disease-resistant plants, thereby enhancing plant productivity (Kumar et al., 2017). The lack of adequate genomic data makes it inevitable to understand the role of resistant gene analogs in *D. sissoo* plants. In this research study (a part of PARB project# 952), we *in silico* characterized the short reads of nucleotide sequences or short-read resistance gene analogs (RGAs) identified in dieback stress-induced transcriptomic analysis for elucidating their role and contribution to dieback resistance in *D. sissoo*.

## 2 Materials and methods

*In silico* characterization of identified short-read DNA sequences was achieved with the aid of computational biology. These RGAs were of short nucleotide sequence length and expressed only in dieback-resistant *D. sissoo* plants. These differentially expressed short-read nucleotide sequences were identified in dieback stress-induced transcriptomic analysis (Ijaz et al., 2022). Primer detail is given in **Supplementary Table S1**.

### 2.1 Motif scanning and protein fingerprints

The translated protein sequences of identified short-sequence RGAs were generated using the Expasy translate tool. ScanProsite program (Hulo et al., 2006) and NCBI-CDD database (Marchler-Bauer et al., 2015) were used to scan protein motifs. However, the PRINTS database (Attwood et al., 2012) was used to analyze the fingerprints of proteins.

### 2.2 Subcellular localization and physicochemical characterization

Subcellular localization was determined by the DeepLoc.2.0 server (Thumuluri et al., 2022). The Protparam web tool (Gasteiger et al., 2005) was used to analyze the physiochemical characteristics of the deduced protein sequences of identified RGAs. The location of protein segments and topology were determined by DeepTMHMM (Hallgren et al., 2022).

### 2.3 Structural annotation and protein modeling

The secondary structures of translated protein sequences identified from short reads of nucleotide sequences were annotated by the SOPMA online server (Geourjon and Deleage, 1995). Homology modeling was performed using SWISS-MODEL (Waterhouse et al., 2018), PHYRE2 (Kelley et al., 2015) and AlphaFold (Jumper et al., 2021). Electrostatic potential and 3D imaging were determined by PyMOL (Yuan et al., 2017). Ligand docking was made using Hex v. 5.1 (Macindoe et al., 2010). Ramachandran plots were generated to assess model quality using the PROCHECK server (Laskowski et al., 1993).

### 2.4 Sequence analysis and antimicrobial peptide prediction

Translated peptide sequences were *in silico* characterized for antimicrobial peptide properties using web tools in the CAMPR3 database. A sliding window of 20-amino acid residues was used to scan and predict potent AMP stretches within the entire peptide sequence. The net charge, hydrophobic ratio, and Boman index were computed using the APD3 database tool, antimicrobial peptide calculator, and the Ad predictor.

## 3 Results

### 3.1 Structural motif analysis

Understanding the protein motifs provides a deep insight into their biological functions and helps to recognize their role in diverse cellular processes. ScanProsite web server was employed to identify structural motifs and functional residues in the **Ds-DbRcaG-07-Rga1p** (GenBank accession number OQ420429) translated sequence. The identified RGA showed a protein kinase 3 and a casein kinase II (CK2) phosphorylation site involved in the stress-responsive pathway and the survival of cells in plants. **Ds-DbRcaG-09-Rga9p** (GenBank accession number OQ420431) and **Ds-DbRcaG-10-Rga13p** (GenBank accession number OQ420432) were predicted with an *N*-glycosylation site, a protein kinase C (PKC), and a casein kinase II phosphorylation site (CK2_PHOSPHO_SITE). Literature documents that plants’ N-linked glycosylation is involved in growth under stress conditions and adaptive immune activation (Wolfert and Boons, 2013; Nagashima et al., 2018).

Protein kinase C is involved in various cellular processes, such as gene expression regulation and signal transduction, and is important for innate and adaptive immunity (Lim et al., 2015). Moreover, N-linked glycans protect the plant against pathogen invasion (Lin et al., 2020). The translated sequence of identified **Ds-DbRcaG-11-Rga15p** (GenBank accession number OQ420433) revealed a casein kinase II phosphorylation site (CK2_PHOSPHO_SITE) along with a protein kinase C phosphorylation site (PKC), which are the attributes and facets of disease resistance genes. However, no motif scanning hit was found in **Ds-DbRcaG-08-Rga4p** (GenBank accession number OQ420430).

However, a motif that belongs to P-type ATPase aminophospholipid translocases (APLT) was found in **Ds-DbRcaG-07-Rga1p** by MOTIF finder NCBI-CDD search tool. P-4 type ATPases APLTs are involved in the movement of phospholipids across the membrane using ATP. During apoptosis, phosphatidylserine (PS) is released to the external leaflet, and membrane integrity is lost. PS acts as an “eat me” signal and ensures the engulfment of apoptotic cells by phagocytic cells, and phagocytosis thus activates the immune system (Chaurio et al., 2009). However, no motif was found in **Ds-DbRcaG-08-Rga4p**, **Ds-DbRcaG-09-Rga09p**, **Ds-DbRcaG-10-Rga13p**, and **Ds-DbRcaG-11-Rga15p**.

### 3.2 Protein fingerprint analysis

The translated sequences of identified RGAs were analyzed for fingerprints by the PRINTS database (Attwood et al., 2004). The deduced amino acid sequence of **Ds-DbRCaG-07-Rga1p** was predicted with a DISHEVELLED3 fingerprint. This four-element fingerprint codes for the disheveled-3 (DVL-3) protein. DVL protein is a member of a highly conserved superfamily involved in activating the Wnt signaling pathway. Wnt signaling is a crucial mechanism that plays an important role in determining cell fate and is important for immune cell homeostasis (Patel et al., 2019). Wnt signal transduction is initiated when Wnt proteins attach to receptors on the cell surface, also known as frizzled proteins. It will activate the B-catenin signaling functions, which involve several functions like proliferation, differentiation, cell renewal, and apoptosis (Ma et al., 2020). Wnt signaling helps fight against the pathogen attack by strengthening the immune system of macrophages (Jati and Sen, 2019).

The identified translated sequence of **Ds-DbRCaG-09-Rga9p** was predicted with the fingerprint of the highest hit, “IGASERPTASE,” which codes for the IgA-specific endopeptidase (S6) family of serine peptidases. Serine peptidase is a protein-cleaving enzyme that mediates various functions, such as apoptosis, proteolysis, and inflammatory responses, thus protecting plants against phytopathogenic microorganisms (Patel, 2017; Clemente et al., 2019). Moreover, serine protease inhibitors in plants have the potential to defend the plants against biotic stress (Hartl et al., 2011).

The translated protein sequence of **Ds-DbRcaG-10-Rga13p** was assigned the fingerprint “HIGHMOBLTYIY,” which predicted its involvement in the immune response. High mobility group (HMG) proteins are the most abundant nuclear proteins, further divided into three distinct superfamilies: HMGA, HMGB, and HMGN. Each can interact with different chromatin sites and is involved in processes like DNA repair, transcriptional regulation, cell signaling, and apoptosis. Moreover, HMG proteins are involved in abiotic stress responses and plant development (Pedersen and Grasser, 2010). These proteins are also predicted to have various phosphorylation sites, such as methylation and glycosylation (Greis and Hart, 1997). These phosphorylation sites are essential to multiple cellular processes like apoptosis and signaling and are involved in plant growth and development (Park et al., 2012). High mobility group box 2 (HMGB2) provides antimicrobial activity and protection against several infectious microbes and pathogens (Küchler et al., 2013).

### 3.3 Prediction of physicochemical characteristics and subcellular localization

Molecular weight is an important factor in the functional characterization of proteins. ProtParam (https://web.expasy.org/protparam/) is a web-based application that analyzes various properties of a given protein. **Ds-DbRCaG-07-Rga1p** was predicted to have a sequence length of 71-amino acid residues, and the molecular weight was deduced to be 8.06 kDa. The analysis revealed that the identified RGA is a basic protein with an isoelectric point of 10.28. Histone is a basic protein that has a role in several important functions, such as transcription and signal transduction (Donev, 2014). The extinction coefficient of a protein was predicted to be 6,990 M^−1^ cm^−1^, which is essential to determine its concentration in water at a wavelength of 280 nm (Hilario et al., 2017). The instability index was 44.94, predicted as an unstable protein. A high aliphatic index (81.13) revealed that **Ds-DbRCaG-07-Rga1p** is a thermostable protein and can survive under extreme environmental conditions (Mizuguchi et al., 2007). The GRAVY value of a protein is used to measure its hydrophobicity or hydrophilicity. It was determined that the identified protein has a GRAVY value of −0.549, which indicates that the translated protein sequence is hydrophilic.

The translated protein sequence **Ds-DbRCaG-08-Rga4p** was predicted to have a total length of 25 amino acids with a molecular weight of 2.65 kDa. The computational analysis revealed that the identified RGA is a basic protein with an isoelectric point of 11.33. The instability index was 58.29, which indicates that the identified protein is unstable in an experimental medium. A high aliphatic index value of 78.00 suggests that the translated protein sequence is thermophilic and responsible for providing plants’ resistance to adverse environmental conditions (Wang et al., 2015). The GRAVY value (0.168) suggested that the identified protein is hydrophobic. Most thermophilic proteins have hydrophobic amino acids (Gromiha et al., 2013). Plant lectin proteins exhibit thermophilic and hydrophobic properties and play a role in antimicrobial activity. The lectins adhere to carbohydrates on the pathogen’s surface, which can degrade the cell wall and inhibit microbes from binding to host cells (Roberts and Goldstein, 1982; Pusztai and Grant, 1998; Fonseca et al., 2022). Similarly, crambin is another hydrophobic plant protein that belongs to the thionin family (Teeter et al., 1981; García Olmedo et al., 1989). Thionins have antifungal and antibacterial properties and a role in plant defense mechanisms (Bohlmann and Broekaert, 1994).

It was predicted that the translated protein sequence **Ds-DbRCaG-09-Rga9p** comprises 53-amino acid residues and has a molecular weight of 5.80 kDa. The isoelectric value (8.14) revealed that the composition of the given protein is slightly basic. The aliphatic index was computed to be 46.04, indicating that the protein is thermally stable, while the computed instability index with a value of 48.92 revealed it to be unstable in an experimental medium. However, the computed extinction coefficient value was 8480 M^−1^ cm^−1^. The GRAVY score was determined to be −0.904, which predicted that the identified protein is hydrophilic.

**Ds-DbRcaG-10-Rga13p** was predicted with a molecular weight of 11.19 kDa and a sequence length of 102 amino acids. The theoretical p*I* was predicted to be 10.10, suggesting that the identified protein is basic. The instability index value of 42.52 indicates that the identified protein is unstable, while the extinction coefficient was 2,980 M^−1^ cm^−1^. A high aliphatic index value (78.33) implies that the identified protein is thermophilic and has the property of functioning at high temperatures (Sterpone and Melchionna, 2012). The identified protein was predicted to have a GRAVY value of −0.392 and is a hydrophilic protein.

The translated protein sequence **Ds-DbRcaG-11-Rga15p** comprises 33-amino acid residues with a molecular weight of 3.81 kDa. The isoelectric point was 11.71, indicating that it is a basic protein. Moreover, the instability index and aliphatic index values were computed at 67.14 and 53.03, respectively. The aliphatic index (53.03) predicted it as thermostable; however, the instability index was predicted as an unstable protein in an experimental medium. The GRAVY score (−0.821) suggested that the identified protein is hydrophilic and can survive in extreme environmental conditions.

The GRAVY score of Ds-DbRCaG-07-Rga1p, Ds-DbRCaG-09-Rga9p, Ds-DbRcaG-10-Rga13p, and Ds-DbRcaG-11-Rga15p predicted them as hydrophilic proteins except for Ds-DbRCaG-08-Rga4p. Late embryogenesis-abundant (LEA) proteins in plants are hydrophilic and can tolerate stress conditions such as desiccation (Chakrabortee et al., 2007). Similarly, another protein, plasma membrane-associated calcium-binding protein (PCaP1), is a hydrophilic protein involved in immunity and plant development (Giovannoni et al., 2021).

The translated protein sequences of all short-read DNA sequences predicted their basic nature. The ribosomal proteins are basic and have potential antimicrobial activity (Hurtado-Rios et al., 2022). Similarly, NAC transcription factors are basic proteins that have a role in defense and contribute to plant immunity (Kikuchi et al., 2000; Yuan et al., 2019).

The signal peptide function determines the protein’s translocation and targeting site. Recognizing a protein’s subcellular location is crucial to ascertaining its functional activities. The subcellular localization of the translated protein sequence **Ds-DbRCaG-07-Rga1p** was determined using a DeepLoc-2.0 server (https://services.healthtech.dtu.dk/?DeepLoc-2.0). The highest subcellular signals were found in the nucleus and cytoplasm, with significant values of 0.6423 and 0.5286, respectively. AtUSP protein in *Arabidopsis thaliana* is localized in the nucleus and cytoplasm and is involved in tolerance to cold stress (Melencion et al., 2017). AtUSP protein in *Arabidopsis thaliana* has antifungal activity as it produces reactive oxygen species (ROS) and alters the potential of mitochondria (Park et al., 2017). **Ds-DbRCaG-08-Rga4p** was predicted to be localized in soluble form with a significant score of 0.8589, followed by the extracellular form with a 0.6131 score. Fibrillin is present in the extracellular matrix and is involved in immune and inflammatory pathways (Zeyer & Reinhardt, 2015). **Ds-DbRCaG-10-Rga13p** was reported to be localized in the nucleus and cytoplasm, with a significant score of 0.5645 and 0.5631, respectively. The results suggested cytoplasmic localization in **Ds-DbRCaG-09-Rga9p** and **Ds-DbRCaG-11-Rga15p** with a critical value of 0.5594 and 0.5858, respectively. The cold shock proteins (ARATH2) from *Arabidopsis thaliana* and *Symbiobacterium thermophilum* (SYMTH) were reported to have a cytoplasmic location. Cold shock proteins (CSP) play a role in plant immunity by rescuing cells under stress (Sasaki and Imai, 2012).

Protein topology provides information about the folding and 3D structure of proteins, which are necessary for functional characterization. It is the most effective method for predicting protein segments, whether they are located inside or outside the transmembrane. Protein topology and location of protein segments were predicted using the DeepTMHMM. Therefore, the translated sequences Ds-DbRCaG-1p-Rga1p, Ds-DbRCaG-08-Rga4p, Ds-DbRCaG-09-Rga9p, Ds-DbRCaG-10-Rga13p, and Ds-DbRCaG-11-Rga15p were subjected to DeepTMHMM *in silico* analysis.

The results indicated that segments of predicted protein sequences for **Ds-DbRCaG-1p-Rga1p**, **Ds-DbRCaG-09-Rga9p**, **Ds-DbRCaG-10-Rga13p**, and **Ds-DbRCaG-11-Rga15p** were localized inside the transmembrane, and all have globular protein topology. Actin is a globular protein located inside the membrane and involved in the perception of pathogens like viruses, fungi, and bacteria and the response against abiotic and abiotic factors (Porter & Day, 2016). Hemoglobin is a globular protein inside the red blood cells (RBCs). Hemoglobin at low concentrations has lethal bactericidal activity (Hobson et al., 1958). However, the segments of the predicted protein sequence of **Ds-DbRCaG-08-Rga4p** were localized both outside and inside the transmembrane. All had globular protein topology. Insulin is a globular protein with receptors inside and outside the transmembrane. Insulin indirectly contributes to antimicrobial activity and oxidative stress (Hegazy et al., 2021). Insulin has anti-inflammatory activity because it can differentiate polarity in immune cells as insulin receptors are present on immune cells. Therefore, insulin copes with immunity during infection (van Niekerk et al., 2020). The predicted topology of deduced amino acid sequences of identified RGAs to actin-like proteins, hemoglobin, and insulin intimated their roles in disease defense mechanisms.

### 3.4 Protein secondary structure annotation

Protein function is crucial to predicting its specific structure. We used the protein structure analysis tool to understand the role of the identified RGAs in resistance against diseases. The self-optimized predicted method with alignment (SOPMA) will forecast the secondary structure of the protein in comparison to the query sequence. The results detected α-helix (Hh), random coil (Cc), β-turn (Tt), and extended strand (Ee) proportions in the translated protein sequence of the identified RGAs. The 9.86% Hh, 21.13% Ee, and 5.63% Tt with a significant share of 63.38% of Cc, were predicted in **Ds-DbRCaG-07-Rga1p**. **Ds-DbRCaG-08-Rga4p** was predicted with 28.21% Ee and 2.56% Hh while Cc contributes a considerable share (69.23).

**Ds-DbRCaG-09-Rga9p** was predicted with 11.32% Ee, 24.53% Hh, and 1.89% Tt, with Cc contributing the significant share (62.26%). **Ds-DbRCaG-10-Rga13p** was predicted with 31.37% Ee, 15.69% Hh, and 3.92% Tt, with Cc contributing the major share (49.02%). **Ds-DbRCaG-11-Rga15p** revealed that Cc accounts for the majority (77.14%), Ee accounts for 17.14%, and Tt and Hh each contribute 2.68% of the total share.

### 3.5 Homology modeling

**Figure 1:**
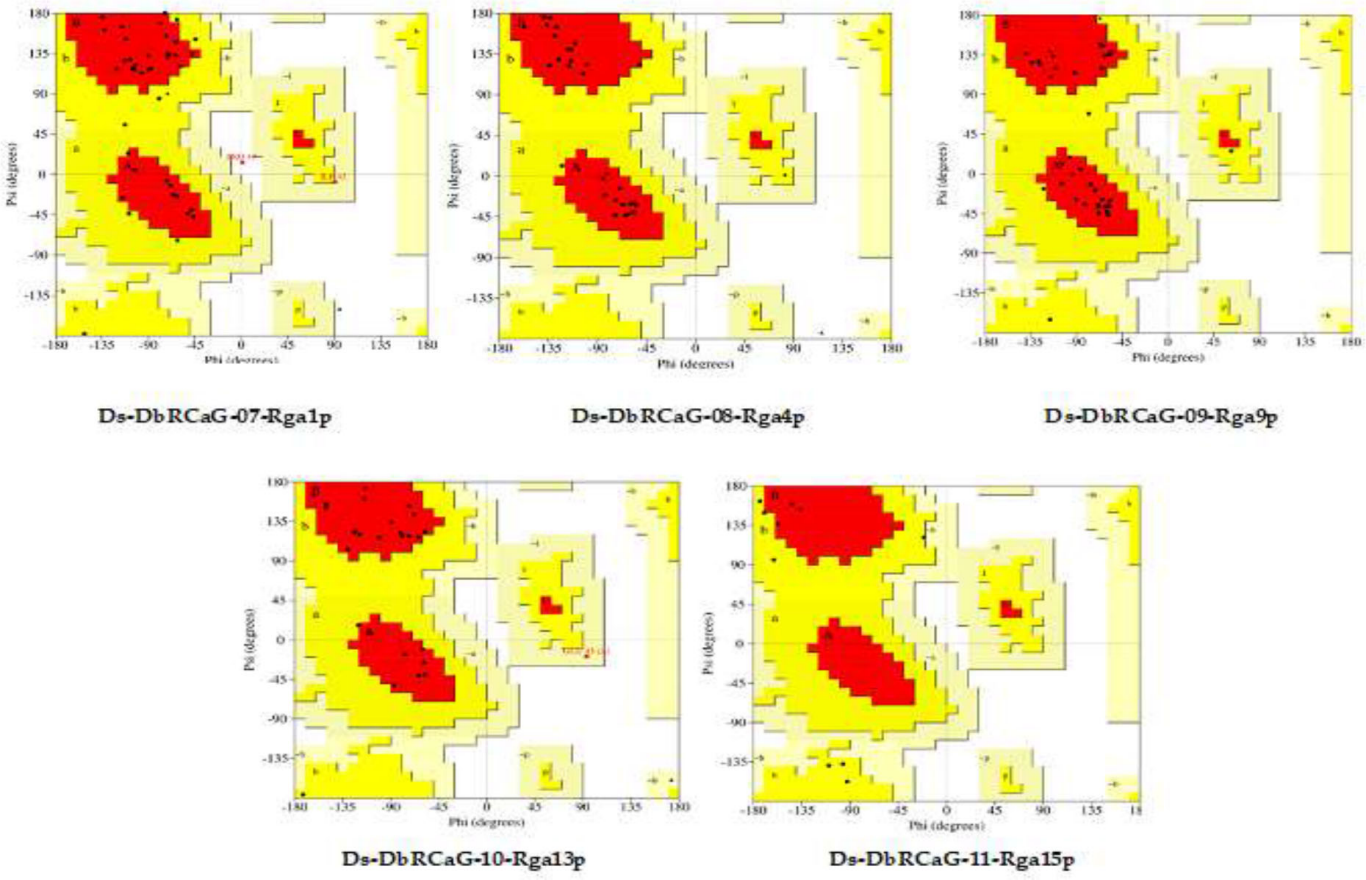
Ramachandran plots of Ds-DbRCaG-07-Rga1p, Ds-DbRCaG-08-Rga4p, Ds-DbRCaG-09-Rga9p, Ds-DbRCaG-10-Rga13p, and Ds-DbRCaG-11-Rga15p. The most favored region is shown in red, the additionally allowed region in yellow, the generously allowed region in pale yellow, and the additionally allowed region in white.

The quality of 3D models generated using the Swiss model and Phyre2 web tools was evaluated by constructing Ramachandran plots. The plot is a graphical representation of amino acids in protein sequence according to phi and psi angles. The model quality assessment displayed 77.1%, 96%, 89.2%, 88.5%, and 28.6% residues in the most favored region of Ramachandran plots generated for 3D models of Ds-DbRCaG-08-Rga4p, Ds-DbRCaG-09-Rga9p, Ds-DbRCaG-10-Rga13p, and Ds-DbRCaG-15-Rga15 (**Figure 1**). The Z-score evaluated the geometric features and nativeness of protein structures in a model. The Swiss model was used for calculating QMEAN *Z* values from zero to −4, indicating a good quality model. QMEAN *Z* values of **Ds-DbRCaG-08-Rga4p**, **Ds-DbRCaG-09-Rga9p**, and **Ds-DbRCaG-10-Rga13p** were −1.16, −2.51, and −1.57, respectively, which validated those models of high quality. The *Z*-score was calculated from the ProSA web server, and a value near −6.57 indicates the model is of good quality. The estimated *Z*-score of **DbRCaG-07-Rga1p** and **DbRCaG-11-Rga15p** were −10.28 and −6.31.

Homology modeling of identified RGAs was predicted through the AlphaFold Protein Structure Database, one of the most effective methods for predicting the 3D structure of proteins. The identified **DbRCaG-07-Rga1p** displayed a similarity with Nicotinamide adenine dinucleotide (NAD) + hydrogen (H)-quinone oxidoreductase subunit H of *Acrobacter* species of bacteria, and **Ds-DbRCaG-08-Rga4p** displayed a homology with NADH-quinone oxidoreductase subunit K of *Sphaerobacter thermophiles*. NADH-quinone oxidoreductase is involved in oxidative stresses (Muras et al., 2016). The NADPH phagocyte is involved in the antimicrobial activity of macrophages against *Salmonella typhimurium* (Vazquez-Torres et al., 2000).

The results in **Ds-DbRCaG-9p-Rga9p** showed a similarity with defensin-like protein A of *Arabidopsis thaliana.* Defensin is a short protein with plant antimicrobial activity (Lay & Anderson, 2005). **Ds-DbRCaG-10-Rga13p** displayed a homology with C-terminal flanking peptide of humans and animals. C-terminal flanking peptides such as defensins are involved in innate immunity in plants against fungi (Lay et al., 2014). **Ds-DbRCaG-11-Rga15p** was similar to the ribonuclease R protein of *Symbiobacterium thermophilum* (SYMTH) and *Pseudomonas* sp. (9PSED). The ribonuclease (RNase) enzyme is reported to have a critical role in stress modulation (Bárria et al., 2016). RNase R is a cold shock protein that helps cells survive in extreme conditions. It was found that RNase R has a role in gene expression and transcriptional regulation in a stationary phase cell (Bárria et al., 2016). Moreover, RNase R activates virulence genes and pathogenesis (Cairrão et al., 2003). These findings predicted their involvement in disease resistance.

**Figure 2:**
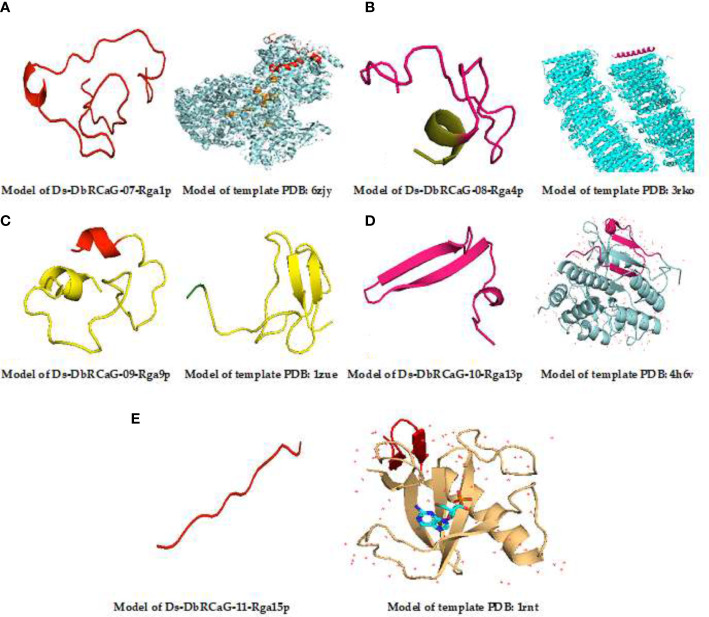
3D homology modeling of Ds-DbRCaG-07-Rga1p, Ds-DbRCaG-08-Rga4p, Ds-DbRCaG-09-Rga9p, Ds-DbRCaG-10-Rga13p, and Ds-DbRCaG-11-Rga15p. The homology regions of the template model and the predicted protein model of each identified RGA are displayed in the same color scheme. **(A)** Predicted protein structure of Ds-DbRCaG-07-Rga1p. **(B)** Predicted protein structure of Ds-DbRCaG-08-Rga4p. **(C)** Predicted protein structure of Ds-DbRCaG-09-Rga9p. **(D)** Predicted protein structure of Ds-DbRCaG-10-Rga13p. **(E)** Protein structure of Ds-DbRCaG-11-Rga15p. The secondary structure model of each template was obtained from the RCSB protein database.

The 3D model of **Ds-DbRCaG-07-Rga1p** displayed a homology to template 6ZJY (crystal structure of respiratory complex I from *Thermus thermophiles*). A total of 16-amino acid residues were modeled with the template. The aligned template region has the NADH quinone oxidoreductase subunit 1 domain connected with the helix. The modeled region was rich in Lys and Glu residues (**Figure 2A**). The 3D model of **Ds-DbRCaG-08-Rga4p** showed a homology to template 3RKO (crystal structure of membrane domain of respiratory complex I from *Escherichia coli*). A total of 29 residues were modeled with the template. The aligned template region has the NADH quinone oxidoreductase subunit 1 domain connected with helices (**Figure 2B**). The 3D model of **Ds-DbRCaG-09-Rga9p** showed a homology to template 4H6V (crystal structure of patellamide maturation protease Pat A from *Escherichia coli*). A total of 31 residues were modeled with the template. The aligned region of the template has the subtilisin-like protein domain connected with α-helices (**Figure 2C**). The 3D model of **Ds-DbRCaG-10-Rga13p** displayed a homology to template 1ZUE (defensin-like peptide 2 toxins).

The 3D model of Ds-DbRCaG-11-Rga15p showed a homology to the 1RNT template (crystal structure of ribonuclease T1 and guanylic complex from *Aspergillus oryzae*). A total of 38 residues were modeled with the template. The aligned region of the template has a DPL2 domain connected with the disulfide bridge. Cys residues were mostly present in the modeled region (**Figure 2D**). A total of 12 residues was modeled with a template having ribonuclease T1 isozyme domain connected with α-helices and extended plates with wide loops, and the modeled region was rich in Cys residues (**Figure 2E**).

### 3.6 Electrostatic potential and docking analysis

**Figure 3:**
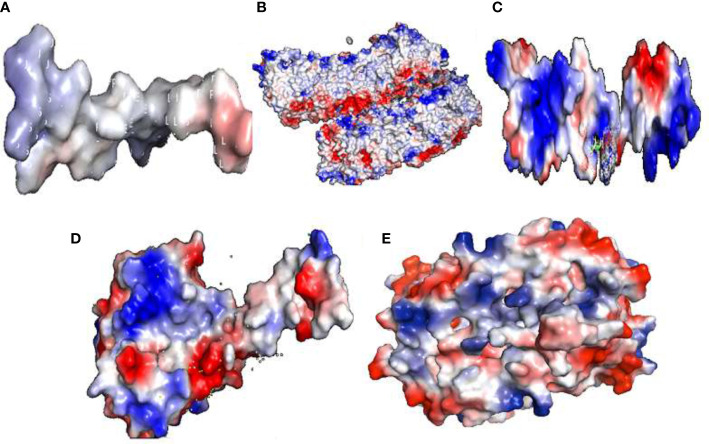
Electrostatic potential: surface electrostatic charge of **(A)** Ds-DbRCaG-07-Rga1p, **(B)** Ds-DbRCaG-08-Rga4p, **(C)** Ds-DbRCaG-09-Rga9p, **(D)** Ds-DbRCaG-10- Rga13p, and **(E)** Ds-DbRCaG-11-Rga15p calculated by PyMOL. Positive charges are shown in the blue region, negative charges in the red region, and neutral charges in the white region.

The electrostatic potential of Ds-DbRCaG-07-Rga1p showed the modeled active region possesses a neutral state (**Figure 3A**). The Ds-DbRCaG-08-Rga4p displayed a charge that was more positive and less neutral (**Figure 3B**). The Ds-DbRCaG-09-Rga9p electrostatic potential showed a modeled domain with a positive charge (**Figure 3C**). The Ds-DbRCaG-10-Rga13p was found to have equal positive and neutral charge states with fewer negative charges (**Figure 3D**). However, the electrostatic potential of Ds-DbRCaG-11-Rga15p fell into a more neutral zone while remaining less positive (**Figure 3E**).

**Figure 4:**
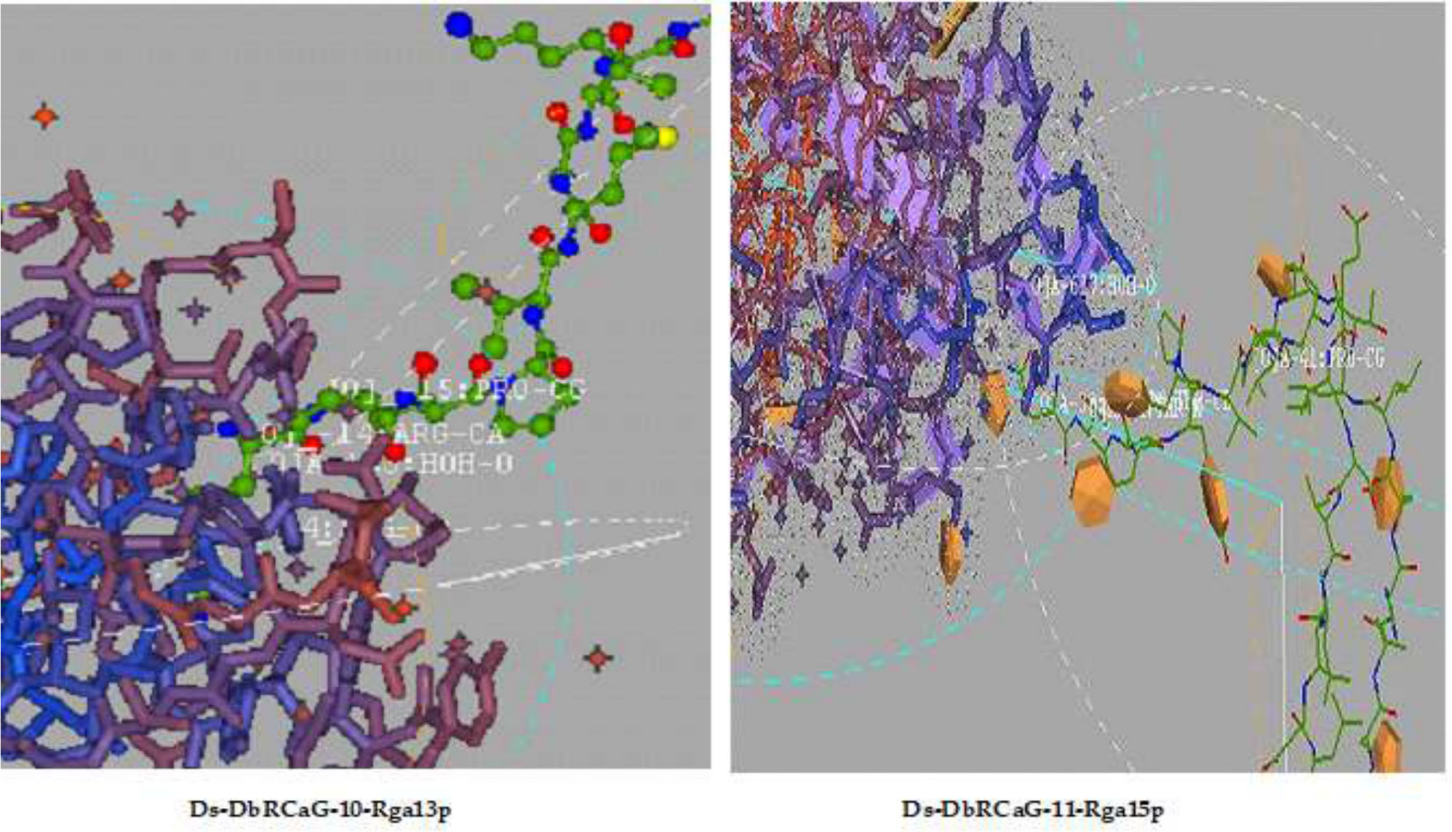
Ligand docking: representative diagrams of Ds-DbRCaG-10-Rga13p and Ds-DbRCaG-11-Rga15p display the active ligand sites in the docked region.

Ligand docking of identified RGAs was performed using Hex 5.1. Ligand-binding sites analyzed in all RGAs were shown to have residues such as Arg, proline (Pro), and Lys (**Figure 4**). The presence of arginine and lysine residues in antimicrobial peptides documented their strong relation with antimicrobial activity (Cutrona et al., 2015). They reported the change in arginine and lysine residues affecting the antimicrobial activity of antimicrobial peptides. However, arginine residues are naturally predominant over lysine residues in antimicrobial peptides (Yeaman and Yount, 2003; Hristova and Wimley, 2011).

### 3.7 Antimicrobial peptide prediction and characterization

The net charge of the translated peptide sequences of all short-read DNA sequences calculated by the APD3 database tool ranged from +1-to +7 with a hydrophobic ratio of 26% to 44% (**Table 1**). These attributes suggested they were putative AMPs. The peptides with a hydrophobic ratio of ≥30% and net charges of +2 to +9 are considered AMPs (Hancock and Sahl, 2006). The peptide with this kind of cationic hydrophobic arrangement imparts an amphipathic (having both hydrophobic and hydrophilic regions) nature to AMPs. This attribute facilitates their interaction with the microbial cell membrane, which is embedded into an anionic membrane and causes microbial death (Semreen et al., 2018). So, the cationic (mainly presence of Lys and Arg amino acids residues) and hydrophobic nature of AMPs is the core attribute of AMPs for their mechanism of action (Arias et al., 2018).

**Table 1:** Physicochemical characteristics and *in silico* prediction of the antimicrobial peptide (AMP) nature of translated peptide sequences from short read nucleotide sequences.

Name	Net charge	Hydrophobic ratio (%)	Boman index (kcal/mol)	CAMP_R3_			
				SVM	RF	ANN	DA
**Ds-DbRCaG-07-Rga1p**	+ 7	32	1.44	NAMP	NAMP	NAMP	NAMP
**Ds-DbRCaG-08-Rga4p**	+ 5	44	0.15	AMP	NAMP	NAMP	AMP
**Ds-DbRCaG-09-Rga9p**	+ 1	26	2.27	NAMP	NAMP	NAMP	NAMP
**Ds-DbRCaG-10-Rga13p**	+ 6	31	1.67	AMP	AMP	NAMP	AMP
**Ds-DbRCaG-11-Rga15p**	+ 4	30	3.33	NAMP	NAMP	NAMP	NAMP

The AMP prediction tool of the CAMPR3 database was used to predict translated peptide sequences of all short-read DNA sequences as potent APMs using four machine learning algorithms: support vector machine (SVM) algorithm, random forest (RF) algorithm, artificial neural network (ANN) algorithm, and discriminant analysis (DA) algorithm. All these algorithms except ANN predict the peptide with the probability of having antimicrobial properties. The probability value “0” indicates low probability, and “1” depicts high probability. A probability value >of 0.5 classified a peptide as active AMP (Hansen et al., 2020). However, the ANN algorithm makes a qualitative statement of either non-AMP (NAMP) or AMP.

The translated peptide sequence of Ds-DbRCaG-08-Rga4p and Ds-DbRCaG-10-Rga13p were predicted as AMPs (**Table 1**). The translated peptide of Ds-DbRCaG-10-Rga13p was predicted to be AMP by SVM, RF, and DA models with a probability value of 1.000, 0.9315, and 0.999, respectively. However, the translated peptide of Ds-DbRCaG-08-Rga4p was predicted to be AMP by SVM and DA models with probability values of 0.523 and 0.767, respectively. However, these algorithms predicted Ds-DbRCaG-07-Rga1p, Ds-DbRCaG-09-Rga9p, and Ds-DbRCaG-11-Rga15p as non-AMP (NAMP). Furthermore, the Boman index of all these sequences was calculated, which predicts protein-binding potential. The value >2.48 shows multifunctional peptide-like hormones; however, the value ≤1 depicts potent AMPs possessing few side effects (Boman, 2003). Among these, the translated peptide sequence of Ds-DbRCaG-08-Rga4p was estimated to have a Boman index value of 0.15 kcal/mol, which predicted it to be a potent AMP with fewer side effects. However, the Boman index of the translated peptides of Ds-DbRCaG-07-Rga1p and Ds-DbRCaG-10-Rga13p was 1.44 kcal/mol, and 1.67 kcal/mol, respectively, which are in the middle range and closer to the defined scale for AMPs.

Therefore, we predicted the antimicrobial regions within all translated peptide sequences (**Supplementary Table S2**). Among these, no antimicrobial region was found in the translated peptide of DbRCaG-09-Rga9p, with a hydrophobic ratio of 26% and a net charge of +1. These values do not lie on the physicochemical scales considered for AMPs. Hereafter, the peptide segments predicted to be AMPs by at least two prediction models were selected to analyze their net charge, hydrophobic ratio, and Boman index (**Table 2**).

**Table 2:** A list of selected predicted antimicrobial peptide segments predicted by AMP prediction models within translated peptide sequences.

Position	Sequence	Net charge	Hydrophobic ratio (%)	Boman index (kcal/mol)	CAMP_R3_						
					SVM		RF		DA		ANN
					Class	Probability	Class	Probability	Class	Probability	Class
Ds-DbRCaG-07-Rga1p											
27-46	DYKRGGAPPPIGLLIQITLK	+ 2	35	0.44	–	–	AMP	0.539	AMP	0.591	AMP
28-47	YKRGGAPPPIGLLIQITLKI	+ 3	40	−0.24	AMP	0.566	AMP	0.601	AMP	0.812	AMP
29-48	KRGGAPPPIGLLIQITLKIW	+ 3	45	−0.36	–	–	AMP	0.596	AMP	0.953	AMP
30-49	RGGAPPPIGLLIQITLKIWI	+ 2	50	−0.88	–	–	AMP	0.646	AMP	0.938	AMP
31-50	GGAPPPIGLLIQITLKIWIF	+ 1	55	−1.78	AMP	0.951	AMP	0.548	AMP	0.980	AMP
32-51	GAPPPIGLLIQITLKIWIFH	+ 1.25	55	−1.5	AMP	0.932	AMP	0.538	AMP	0.936	AMP
33-52	APPPIGLLIQITLKIWIFHF	+ 1.25	60	−1.6	AMP	0.899	AMP	0.542	AMP	0.530	AMP
34-53	PPPIGLLIQITLKIWIFHFH	+ 1.5	55	−1.28	AMP	0.599	AMP	0.514	–	–	AMP
35-54	PPIGLLIQITLKIWIFHFHK	+ 2.5	55	−1	AMP	0.760	AMP	0.562	AMP	0.819	AMP
36-55	PIGLLIQITLKIWIFHFHKI	+ 2.5	60	−1.24	AMP	0.861	AMP	0.634	AMP	0.966	AMP
37-56	IGLLIQITLKIWIFHFHKIH	+ 2.75	60	−1.01	AMP	0.976	AMP	0.731	AMP	0.993	AMP
38-57	GLLIQITLKIWIFHFHKIHL	+ 2.75	60	−1.01	AMP	0.955	AMP	0.720	AMP	0.991	AMP
39-58	LLIQITLKIWIFHFHKIHLP	+ 2.75	60	−0.96	AMP	0.870	AMP	0.654	AMP	0.939	AMP
40-59	LIQITLKIWIFHFHKIHLPL	+ 2.75	60	−0.96	AMP	0.840	AMP	0.655	AMP	0.939	AMP
41-60	IQITLKIWIFHFHKIHLPLN	+ 2.75	60	−0.96	AMP	0.826	AMP	0.649	AMP	0.974	AMP
42-61	QITLKIWIFHFHKIHLPLNL	+ 2.75	55	−0.39	AMP	0.581	AMP	0.606	AMP	0.941	AMP
43-62	ITLKIWIFHFHKIHLPLNLH	+ 3	55	−0.43	AMP	0.766	AMP	0.661	AMP	0.934	AMP
44-63	TLKIWIFHFHKIHLPLNLHL	+ 3	55	−0.43	AMP	0.519	AMP	0.600	AMP	0.898	AMP
45-64	LKIWIFHFHKIHLPLNLHLS	+ 3	55	−0.39	AMP	0.788	AMP	0.666	AMP	0.897	AMP
46-65	KIWIFHFHKIHLPLNLHLSS	+ 3	50	0.02	AMP	0.697	AMP	0.580	AMP	0.930	AMP
47-66	IWIFHFHKIHLPLNLHLSSH	+ 2.25	50	−0.02	AMP	0.710	AMP	0.532	AMP	0.951	AMP
48-67	WIFHFHKIHLPLNLHLSSHF	+ 2.25	50	0.07	AMP	0.605	–	–	AMP	0.901	AMP
49-68	IFHFHKIHLPLNLHLSSHFT	+ 2.25	45	0.31	AMP	0.689	–	–	AMP	0.938	AMP
50-69	FHFHKIHLPLNLHLSSHFTK	+ 3.25	40	0.84	AMP	0.542	AMP	0.509	AMP	0.905	AMP
51-70	HFHKIHLPLNLHLSSHFTKS	+ 3.25	35	1.16	–	–	–	–	AMP	0.776	AMP
Ds-DbRCaG-08-Rga4p											
2-21	IKRMPPFKGKPSAKASPGVF	+ 5	35	1.05	AMP	0.734	–	–	AMP	0.924	AMP
6-25	PPFKGKPSAKASPGVFLLVG	+ 3	40	−0.34	AMP	0.771	AMP	0.672	AMP	0.962	AMP
Ds-DbRCaG-10-Rga13p											
2-21	NFSDTSSFKFLRTKGSIGHA	+ 2.25	30	2.11	–	–	–	–	AMP	0.629	AMP
3-22	FSDTSSFKFLRTKGSIGHAF	+ 2.25	35	1.63	–	–	–	–	AMP	0.615	AMP
6-25	TSSFKFLRTKGSIGHAFTVR	+ 4.25	35	1.85	–	–	AMP	0.556	AMP	0.846	AMP
7-26	SSFKFLRTKGSIGHAFTVRI	+ 4.25	40	1.47	0.517	0.517	AMP	0.705	AMP	0.931	AMP
8-27	SFKFLRTKGSIGHAFTVRIR	+ 5.25	40	2.05	–	–	AMP	0.764	AMP	0.960	AMP
9-28	FKFLRTKGSIGHAFTVRIRT	+ 5.25	40	2.01	AMP	0.702	AMP	0.894	AMP	0.937	AMP
10-29	KFLRTKGSIGHAFTVRIRTG	+ 5.25	35	2.11	AMP	0.675	AMP	0.867	AMP	0.962	AMP
11-30	FLRTKGSIGHAFTVRIRTGN	+ 4.25	35	2.16	AMP	0.590	AMP	0.826	AMP	0.922	AMP
12-31	LRTKGSIGHAFTVRIRTGNQ	+ 4.25	30	2.59	–	–	AMP	0.551	AMP	0.521	NAMP
39-58	FVPHEISVLVELILGHLRYL	− 0.5	55	-0.45	–	–	AMP	0.577	AMP	0.515	NAMP
72-91	NVFRPDRPAEAGLGSKKRGG	+ 3	25	3.1	–	–	AMP	0.508	AMP	0.729	AMP
73-92	VFRPDRPAEAGLGSKKRGGA	+ 3	30	2.68	–	–	AMP	0.566	AMP	0.895	AMP
74-93	FRPDRPAEAGLGSKKRGGAP	+ 3	25	2.88	–	–	–	–	AMP	0.514	AMP
80-99	AEAGLGSKKRGGAPPPIHGI	+ 2.25	30	0.8	–	–	AMP	0.529	AMP	0.711	AMP
81-100	EAGLGSKKRGGAPPPIHGIS	+ 2.25	25	1.06	AMP	0.604	AMP	0.545	AMP	0.548	AMP
82-101	AGLGSKKRGGAPPPIHGISK	+ 4.25	25	0.99	AMP	0.798	AMP	0.858	AMP	0.965	AMP
83-102	GLGSKKRGGAPPPIHGISKI	+ 4.25	25	0.84	AMP	0.869	AMP	0.949	AMP	0.989	AMP

We searched the homology of predicted AMPs against the signature domains of AMP families and gene families using the CAMPR3 database. We found that predicted AMPs have similarities with the signature region of the PR-5-like gene family and AMP family Thaumatin except for six AMPs. Thus, these predicted AMP sequences were named **DsAMP_07-1p_-1** (27-46, DYKRGGAPPPIGLLIQITLK), **DsAMP_07-1p_-2** (28-47, YKRGGAPPPIGLLIQITLKI), **DsAMP_07-1p_-3** (29-48, KRGGAPPPIGLLIQITLKIW), **DsAMP_07-1p_-4** (30-49, RGGAPPPIGLLIQITLKIWI), **DsAMP_07-1p_-5** (31-50, GGAPPPIGLLIQITLKIWIF), **DsAMP_07-1p_-6** (32-51, GAPPPIGLLIQITLKIWIFH), **DsAMP_07-1p_-7** (33-52, APPPIGLLIQITLKIWIFHF), **DsAMP_07-1p_-8** (34-53, PPPIGLLIQITLKIWIFHFH), **DsAMP_07-1p_-9** (35-54, PPIGLLIQITLKIWIFHFHK), **DsAMP_10-13p_-1** (10-29, KFLRTKGSIGHAFTVRIRTG), **DsAMP_10-13p_-2** (11-30, FLRTKGSIGHAFTVRIRTGN), **DsAMP_10-13p_-3** (12-31, LRTKGSIGHAFTVRIRTGNQ), **DsAMP_10-13p_-4** (80-99, AEAGLGSKKRGGAPPPIHGI), **DsAMP_10-13p_-5** (81-100, EAGLGSKKRGGAPPPIHGIS), **DsAMP_10-13p_-6** (82-101, AGLGSKKRGGAPPPIHGISK), and **DsAMP_10-13p_-7** (83-102, GLGSKKRGGAPPPIHGISKI). The underlined regions of predicted AMPs showed a homology with the signature regions of PR-5-like protein. The pathogenesis-related gene family or PR-gene family is involved in defense against pathogens. The PR genes are induced and activated in systemic acquired resistance. Thaumatin-like proteins (TLPs) are a complex protein family that belongs to the PR-5 gene family in plants, including osmotin-like proteins, osmotin, and permatin. Their biosynthesis triggers mainly against biotic stresses (Sharma et al., 2022).

However, the six AMPs showed homology with the Cathelicidin family. These were named hereafter **DsAMP_8-4p_-1** (2-21, IKRMPPFKGKPSAKASPGVF), **DsAMP_8-4p_-2** (6-25, PPFKGKPSAKASPGVFLLVG), **DsAMP_10-13p_-8** (39-58, FVPHEISVLVELILGHLRYL), **DsAMP_10-13p_-9** (72-91, NVFRPDRPAEAGLGSKKRGG), **DsAMP_10-13p_-10** (73-92, VFRPDRPAEAGLGSKKRGGA), and **DsAMP_10-13p_-11** (74-93, FRPDRPAEAGLGSKKRGGAP). The Cathelicidin family is one of the major groups of AMPs in humans and plants (Jung and Kang, 2014). Cathelicidins are antimicrobial peptides having a wide range of activity against bacteria, fungi, and viruses. They kill microbes directly or by binding to the endotoxin (Ramanathan et al., 2002). Cathelicidins are the components of the innate immune system, providing rapid responses to pathogens (Roby and Di Nardo, 2013).

## 4 Discussion

Plants are sessile, so they have to develop the best immune system against biotic and abiotic stresses. Plant–pathogen interaction occurs between pathogens and molecules such as lipopolysaccharides, proteins, and plants’ sugars. The first interaction occurs in the apoplast with the identification of elicitors of microbes by plant receptors. Elicitors are also termed PAMPs. These are detected by the plant membrane-localized PRRs. The first line of defense induced by PAMPs and PRRs is PAMP-triggered immunity (PTI). The second line of defense is activated when resistance proteins (R) counter the effect of effector proteins, and this interaction is known as effector-triggered immunity (ETI). ETI can cause localized cell death (hypersensitive response) in plants infected with pathogens and pathogens. It is stronger than PTI (Gupta et al., 2015). When the plant resistance locus interacts with the pathogen’s avirulence (avr) gene, signal cascading is generated, which activates the plant’s immune system and kills the pathogen (Dangl and Jones, 2001).

Ds-DbRCaG-07-Rga1p displayed a homology with the NADH-quinone oxidoreductase subunit H, and Ds-DbRCaG-08-Rga04 displayed a homology with the NADH-quinone oxidoreductase subunit K. High levels of NADH increase the concentration of ROS in the cell. Increased levels of ROS are involved in the antimicrobial activity (Arce-Rodríguez et al., 2022). Ds-DbRCaG-09-Rga9p showed similarity with elongation factor P and defensin-like protein A. Plant defensins are highly stable, cysteine-rich, and have small peptides involved in innate immunity. Plant defensins are involved in antifungal and antibacterial activity. Most plant defensins are involved in defense against a wide spectrum of fungi, such as phytopathogenic fungi (Stotz et al., 2009). Ds-DbRCaG-10-Rga13p displayed a homology with C-terminal flanking peptides such as defensins. Defensins are antimicrobial peptides involved in the killing of microbes such as fungi. Defensins interact with the plasma membrane of fungi, causing leakage of ions, producing ROS, and ultimately causing the death of fungi (Sher Khan et al., 2019).

Ds-DbRCaG-11-Rga15p found a similarity with the ribonuclease R protein. Pathogenesis-related proteins (PR) such as PR4 and PR10 have ribonuclease activity. The ribonuclease activity of PR proteins is related to antifungal activity. The mechanism of PR proteins includes both cytotoxic effects on cells directly and apoptosis of infected areas of plant cells developing hypersensitive reaction (HR) (Filipenko et al., 2013). P4-type ATPase aminophospholipid translocases (APLT) motif in Ds-DbRCaG-07-Rga1p was found in the MOTIF web tool. P4-type ATPase aminophospholipid translocases transport phospholipids across the plasma membrane, thus maintaining the lipid asymmetry of the membrane. They are involved in cell signaling, cell trafficking, and apoptosis (Andersen et al., 2016).

The PRINTS database predicted Dishevelled 3 fingerprints for Ds-DbRCaG-07-Rga1p, revealing its strong role in signaling pathways. A previous study has also reported that Dishevelled proteins contain phosphorylation sites, which showed that they are involved in gene expression and signaling (Garcia-Garcia et al., 2016). Ds-DbRCaG-09-Rga9p was anticipated to contain a fingerprint IGASERPTASE of the serine peptidase family, which is reported to have a role in physiological functions and defense mechanisms. Serine proteases in potato tubers have antifungal and antibacterial activity (Kim et al., 2009). In plants, protease inhibitors are involved in defense mechanisms against phytopathogenic organisms. Ds-DbRCaG-10-Rga13p displayed a “HIGHMOBLTYIY” fingerprint, indicating it is an important regulator of gene expression. HMG protein (OsHMGB707) in rice is reportedly tolerant against drought stress (Xu et al., 2021).

Using ProtParam, we determined the physiochemical characteristics of the identified RGAs. The results revealed that Ds-DbRCaG-07-Rga1p, Ds-DbRCaG-09-Rga9p, Ds-DbRCaG-10-Rga13p, and Ds-DbRCaG-11-Rga15p are basic proteins. Eosinophil protein (MBP) has antibacterial activity against microbes (Lehrer et al., 1989). Arginine has an essential role in immune mechanisms and in combating pathogen attacks. Moreover, it also provides resistance against abiotic and biotic stress conditions (Gogoi et al., 2016). Ds-DbRCaG-08-Rga4p was reported to be an acidic protein. Most PR1-type proteins in plants are acidic. During infection, they change the ion flux, causing the production of reactive oxygen species and resulting in the death of cells at the infection site (Stolzenberg et al., 2017).

Translated peptide sequences were characterized as having antimicrobial attributes using the CAMPR3 database with 10,247 conserved AMP sequence signatures (Waghu et al., 2016). Among AMP prediction tools, CAMPR3 tools outperform others for predicting AMPs. Though, among CAMPR3 tools, the SVM algorithm-based model is the best performer for AMP prediction (Gabere and Noble, 2017). The net charge of the translated peptide sequences of all short-read DNA sequences calculated by the APD3 database tool ranged from +1 to +7 with a hydrophobic ratio of 26% to 44% (**Table 1**). These attributes suggested they were putative AMPs. The peptides with a hydrophobic ratio of ≥30% and net charges of +2 to +9 are considered AMPs (Hancock and Sahl, 2006). The peptide with this kind of cationic hydrophobic arrangement imparts an amphipathic (having both hydrophobic and hydrophilic regions) nature to AMPs. This attribute facilitates their interaction with microbial cell membranes, and due to that, they are embedded into anionic membranes and cause microbial death (Semreen et al., 2018). Thus, the cationic (mainly presence of Lys and Arg amino acids residues) and hydrophobic nature of AMPs is the core attribute of AMPs for their mechanism of action (Arias et al., 2018). The predicted AMPs have similarities with the signature regions of the PR-5-like gene family and the AMP family Thaumatin. Thaumatin-like proteins belong to the PR-5 gene family in plants. They include osmotin-like proteins, osmotin, and permatin, and their biosynthesis triggers mainly against biotic stresses (Iqbal et al., 2020; Sharma et al., 2022).

## Data availability statement

The original contributions presented in the study are publicly available. This data can be found here: NCBI GenBank, accession numbers OQ420429, OQ420430, OQ420431, OQ420432, OQ420433.

## Author contributions

Conceptualization: SI and IH. Data curation: SI and IH. Formal analysis: SI, IH, RM and GN. Funding acquisition: SI. Investigation: SI and IH. Methodology: SI and IH. Project administration: SI. Resources, SI, IH and HA. Software: SI, IH, RM and GN. Supervision: SI. Validation: SI, IH and SK. Visualization: SI. Writing—original draft: SI, IH, RM and GN. Writing—review and editing: SI, IH, HA and SK. All authors contributed to the article and approved the submitted version.
