# Supplementary material for: In silico characterization of differentially expressed short-read nucleotide sequences identified in dieback stress-induced transcriptomic analysis reveals their role as antimicrobial peptides
Source: Front Plant Sci. 2023 Mar 20;14:1168221. doi: 10.3389/fpls.2023.1168221 (PMC10069654; doi:10.3389/fpls.2023.1168221)
Supplement: Supplementary file 1 [file Table_1.docx]

**Table S1:** Degenerate primers of NBS domain used to recover putative resistance gene sequences or RGAs in shisham upregulated against dieback disease.

| *Degenerate primer* | *Primer sequence (5′-3′)* | *References* |
| --- | --- | --- |
| dgPL-a1F | GGNGGNRTNGGNAAGACGAC | (Noir et al., 2001) |
| dgPL-a2F | GGNGGNRTIGGIAARACIAC | (Sun et al., 2010) |
| dgPL-a3F | GGIGGIGTIGGIAAIACIAC | (Leister et al., 1996) |
| dgPL-a4F | TGSSRGGHWYRGGBAAAACTAC | (Zhang et al., 2008) |
| dgPL-a5F | GGTGGGGTTGGGAAGACAACG | (Leister et al., 1996) |
| dgGL-b1R | GAGGGCTAAAGGAAGGCC | (Deng et al., 2000) |
| dgGL-b2R | IAGIGCIAGIGGIAGICC | (Leister et al., 1996) |
| dgGL-b3R | AAGIGCTAAGIGGIAAGICC | (Peraza-Echeverria et al., 2008) |
| dgGL-b4R | HRCWARAGGVARCCCTYBACA | (Naresh et al., 2017). |
| dgGL-b5R | GAGGGCNARNGGNAAICC | (Noir et al., 2001) |

**Table S2**

**Predicted Antimicrobial region with translated peptide sequence of Ds-DbRCaG-07-Rga1p**

**Results with Support Vector Machine (SVM) classifier**

| **Seq. ID.** | **Position** | **Sequence** | **Class** | **AMP Probability** |
| --- | --- | --- | --- | --- |
| 1 | 28-47 | YKRGGAPPPIGLLIQITLKI | AMP | 0.566 |
| 1 | 31-50 | GGAPPPIGLLIQITLKIWIF | AMP | 0.951 |
| 1 | 32-51 | GAPPPIGLLIQITLKIWIFH | AMP | 0.932 |
| 1 | 33-52 | APPPIGLLIQITLKIWIFHF | AMP | 0.899 |
| 1 | 34-53 | PPPIGLLIQITLKIWIFHFH | AMP | 0.599 |
| 1 | 35-54 | PPIGLLIQITLKIWIFHFHK | AMP | 0.760 |
| 1 | 36-55 | PIGLLIQITLKIWIFHFHKI | AMP | 0.861 |
| 1 | 37-56 | IGLLIQITLKIWIFHFHKIH | AMP | 0.976 |
| 1 | 38-57 | GLLIQITLKIWIFHFHKIHL | AMP | 0.955 |
| 1 | 39-58 | LLIQITLKIWIFHFHKIHLP | AMP | 0.870 |
| 1 | 40-59 | LIQITLKIWIFHFHKIHLPL | AMP | 0.840 |
| 1 | 41-60 | IQITLKIWIFHFHKIHLPLN | AMP | 0.826 |
| 1 | 42-61 | QITLKIWIFHFHKIHLPLNL | AMP | 0.581 |
| 1 | 43-62 | ITLKIWIFHFHKIHLPLNLH | AMP | 0.766 |
| 1 | 44-63 | TLKIWIFHFHKIHLPLNLHL | AMP | 0.519 |
| 1 | 45-64 | LKIWIFHFHKIHLPLNLHLS | AMP | 0.788 |
| 1 | 46-65 | KIWIFHFHKIHLPLNLHLSS | AMP | 0.697 |
| 1 | 47-66 | IWIFHFHKIHLPLNLHLSSH | AMP | 0.710 |
| 1 | 48-67 | WIFHFHKIHLPLNLHLSSHF | AMP | 0.605 |
| 1 | 49-68 | IFHFHKIHLPLNLHLSSHFT | AMP | 0.689 |
| 1 | 50-69 | FHFHKIHLPLNLHLSSHFTK | AMP | 0.542 |

**Results with Random Forest Classifier**

| **Seq. ID.** | **Position** | **Sequence** | **Class** | **AMP Probability** |
| --- | --- | --- | --- | --- |
| 1 | 16-35 | AQQKPENAGHSDYKRGGAPP | AMP | 0.511 |
| 1 | 17-36 | QQKPENAGHSDYKRGGAPPP | AMP | 0.589 |
| 1 | 27-46 | DYKRGGAPPPIGLLIQITLK | AMP | 0.539 |
| 1 | 28-47 | YKRGGAPPPIGLLIQITLKI | AMP | 0.601 |
| 1 | 29-48 | KRGGAPPPIGLLIQITLKIW | AMP | 0.596 |
| 1 | 30-49 | RGGAPPPIGLLIQITLKIWI | AMP | 0.646 |
| 1 | 31-50 | GGAPPPIGLLIQITLKIWIF | AMP | 0.548 |
| 1 | 32-51 | GAPPPIGLLIQITLKIWIFH | AMP | 0.538 |
| 1 | 33-52 | APPPIGLLIQITLKIWIFHF | AMP | 0.542 |
| 1 | 34-53 | PPPIGLLIQITLKIWIFHFH | AMP | 0.514 |
| 1 | 35-54 | PPIGLLIQITLKIWIFHFHK | AMP | 0.562 |
| 1 | 36-55 | PIGLLIQITLKIWIFHFHKI | AMP | 0.634 |
| 1 | 37-56 | IGLLIQITLKIWIFHFHKIH | AMP | 0.731 |
| 1 | 38-57 | GLLIQITLKIWIFHFHKIHL | AMP | 0.720 |
| 1 | 39-58 | LLIQITLKIWIFHFHKIHLP | AMP | 0.654 |
| 1 | 40-59 | LIQITLKIWIFHFHKIHLPL | AMP | 0.655 |
| 1 | 41-60 | IQITLKIWIFHFHKIHLPLN | AMP | 0.649 |
| 1 | 42-61 | QITLKIWIFHFHKIHLPLNL | AMP | 0.606 |
| 1 | 43-62 | ITLKIWIFHFHKIHLPLNLH | AMP | 0.661 |
| 1 | 44-63 | TLKIWIFHFHKIHLPLNLHL | AMP | 0.600 |
| 1 | 45-64 | LKIWIFHFHKIHLPLNLHLS | AMP | 0.666 |
| 1 | 46-65 | KIWIFHFHKIHLPLNLHLSS | AMP | 0.580 |
| 1 | 47-66 | IWIFHFHKIHLPLNLHLSSH | AMP | 0.532 |
| 1 | 50-69 | FHFHKIHLPLNLHLSSHFTK | AMP | 0.509 |

**Results with Artificial Neural Network (ANN) classifier**

| **Seq. ID.** | **Position** | **Sequence** | **Class** |
| --- | --- | --- | --- |
| 1 | 22-41 | NAGHSDYKRGGAPPPIGLLI | AMP |
| 1 | 23-42 | AGHSDYKRGGAPPPIGLLIQ | AMP |
| 1 | 24-43 | GHSDYKRGGAPPPIGLLIQI | AMP |
| 1 | 27-46 | DYKRGGAPPPIGLLIQITLK | AMP |
| 1 | 28-47 | YKRGGAPPPIGLLIQITLKI | AMP |
| 1 | 29-48 | KRGGAPPPIGLLIQITLKIW | AMP |
| 1 | 30-49 | RGGAPPPIGLLIQITLKIWI | AMP |
| 1 | 31-50 | GGAPPPIGLLIQITLKIWIF | AMP |
| 1 | 32-51 | GAPPPIGLLIQITLKIWIFH | AMP |
| 1 | 33-52 | APPPIGLLIQITLKIWIFHF | AMP |
| 1 | 34-53 | PPPIGLLIQITLKIWIFHFH | AMP |
| 1 | 35-54 | PPIGLLIQITLKIWIFHFHK | AMP |
| 1 | 36-55 | PIGLLIQITLKIWIFHFHKI | AMP |
| 1 | 37-56 | IGLLIQITLKIWIFHFHKIH | AMP |
| 1 | 38-57 | GLLIQITLKIWIFHFHKIHL | AMP |
| 1 | 39-58 | LLIQITLKIWIFHFHKIHLP | AMP |
| 1 | 40-59 | LIQITLKIWIFHFHKIHLPL | AMP |
| 1 | 41-60 | IQITLKIWIFHFHKIHLPLN | AMP |
| 1 | 42-61 | QITLKIWIFHFHKIHLPLNL | AMP |
| 1 | 43-62 | ITLKIWIFHFHKIHLPLNLH | AMP |
| 1 | 44-63 | TLKIWIFHFHKIHLPLNLHL | AMP |
| 1 | 45-64 | LKIWIFHFHKIHLPLNLHLS | AMP |
| 1 | 46-65 | KIWIFHFHKIHLPLNLHLSS | AMP |
| 1 | 47-66 | IWIFHFHKIHLPLNLHLSSH | AMP |
| 1 | 48-67 | WIFHFHKIHLPLNLHLSSHF | AMP |
| 1 | 49-68 | IFHFHKIHLPLNLHLSSHFT | AMP |
| 1 | 50-69 | FHFHKIHLPLNLHLSSHFTK | AMP |
| 1 | 51-70 | HFHKIHLPLNLHLSSHFTKS | AMP |

**Results with Discriminant Analysis classifier**

| **Seq. ID.** | **Position** | **Sequence** | **Class** | **AMP Probability** |
| --- | --- | --- | --- | --- |
| 1 | 27-46 | DYKRGGAPPPIGLLIQITLK | AMP | 0.591 |
| 1 | 28-47 | YKRGGAPPPIGLLIQITLKI | AMP | 0.812 |
| 1 | 29-48 | KRGGAPPPIGLLIQITLKIW | AMP | 0.953 |
| 1 | 30-49 | RGGAPPPIGLLIQITLKIWI | AMP | 0.938 |
| 1 | 31-50 | GGAPPPIGLLIQITLKIWIF | AMP | 0.980 |
| 1 | 32-51 | GAPPPIGLLIQITLKIWIFH | AMP | 0.936 |
| 1 | 33-52 | APPPIGLLIQITLKIWIFHF | AMP | 0.530 |
| 1 | 35-54 | PPIGLLIQITLKIWIFHFHK | AMP | 0.819 |
| 1 | 36-55 | PIGLLIQITLKIWIFHFHKI | AMP | 0.966 |
| 1 | 37-56 | IGLLIQITLKIWIFHFHKIH | AMP | 0.993 |
| 1 | 38-57 | GLLIQITLKIWIFHFHKIHL | AMP | 0.991 |
| 1 | 39-58 | LLIQITLKIWIFHFHKIHLP | AMP | 0.939 |
| 1 | 40-59 | LIQITLKIWIFHFHKIHLPL | AMP | 0.939 |
| 1 | 41-60 | IQITLKIWIFHFHKIHLPLN | AMP | 0.974 |
| 1 | 42-61 | QITLKIWIFHFHKIHLPLNL | AMP | 0.941 |
| 1 | 43-62 | ITLKIWIFHFHKIHLPLNLH | AMP | 0.934 |
| 1 | 44-63 | TLKIWIFHFHKIHLPLNLHL | AMP | 0.898 |
| 1 | 45-64 | LKIWIFHFHKIHLPLNLHLS | AMP | 0.897 |
| 1 | 46-65 | KIWIFHFHKIHLPLNLHLSS | AMP | 0.930 |
| 1 | 47-66 | IWIFHFHKIHLPLNLHLSSH | AMP | 0.951 |
| 1 | 48-67 | WIFHFHKIHLPLNLHLSSHF | AMP | 0.901 |
| 1 | 49-68 | IFHFHKIHLPLNLHLSSHFT | AMP | 0.938 |
| 1 | 50-69 | FHFHKIHLPLNLHLSSHFTK | AMP | 0.905 |
| 1 | 51-70 | HFHKIHLPLNLHLSSHFTKS | AMP | 0.776 |
| 1 | 52-71 | FHKIHLPLNLHLSSHFTKSD | AMP | 0.719 |

**Predicted Antimicrobial region with translated peptide sequence of Ds-DbRCaG-08-Rga4p**

**Results with Support Vector Machine (SVM) classifier**

| **Seq. ID.** | **Position** | **Sequence** | **Class** | **AMP Probability** |
| --- | --- | --- | --- | --- |
| 1 | 2-21 | IKRMPPFKGKPSAKASPGVF | AMP | 0.734 |
| 1 | 6-25 | PPFKGKPSAKASPGVFLLVG | AMP | 0.771 |

**Results with Random Forest Classifier**

| **Seq. ID.** | **Position** | **Sequence** | **Class** | **AMP Probability** |
| --- | --- | --- | --- | --- |
| 1 | 6-25 | PPFKGKPSAKASPGVFLLVG | AMP | 0.672 |

**Results with Artificial Neural Network (ANN) classifier**

| **Seq. ID.** | **Position** | **Sequence** | **Class** |
| --- | --- | --- | --- |
| 1 | 2-21 | IKRMPPFKGKPSAKASPGVF | AMP |
| 1 | 6-25 | PPFKGKPSAKASPGVFLLVG | AMP |

**Results with Discriminant Analysis classifier**

| **Seq. ID.** | **Position** | **Sequence** | **Class** | **AMP Probability** |
| --- | --- | --- | --- | --- |
| 1 | 1-20 | MIKRMPPFKGKPSAKASPGV | AMP | 0.768 |
| 1 | 2-21 | IKRMPPFKGKPSAKASPGVF | AMP | 0.924 |
| 1 | 3-22 | KRMPPFKGKPSAKASPGVFL | AMP | 0.631 |
| 1 | 5-24 | MPPFKGKPSAKASPGVFLLV | AMP | 0.501 |
| 1 | 6-25 | PPFKGKPSAKASPGVFLLVG | AMP | 0.962 |

**Predicted Antimicrobial region with translated peptide sequence of Ds-DbRCaG-10-Rga13p**

**Results with Support Vector Machine (SVM) classifier**

| **Seq. ID.** | **Position** | **Sequence** | **Class** | **AMP Probability** |
| --- | --- | --- | --- | --- |
| 1 | 7-26 | SSFKFLRTKGSIGHAFTVRI | AMP | 0.517 |
| 1 | 9-28 | FKFLRTKGSIGHAFTVRIRT | AMP | 0.702 |
| 1 | 10-29 | KFLRTKGSIGHAFTVRIRTG | AMP | 0.675 |
| 1 | 11-30 | FLRTKGSIGHAFTVRIRTGN | AMP | 0.590 |
| 1 | 15-34 | KGSIGHAFTVRIRTGNQNQT | AMP | 0.594 |
| 1 | 81-100 | EAGLGSKKRGGAPPPIHGIS | AMP | 0.604 |
| 1 | 82-101 | AGLGSKKRGGAPPPIHGISK | AMP | 0.798 |
| 1 | 83-102 | GLGSKKRGGAPPPIHGISKI | AMP | 0.869 |

**Results with Random Forest Classifier**

| **Seq. ID.** | **Position** | **Sequence** | **Class** | **AMP Probability** |
| --- | --- | --- | --- | --- |
| 1 | 6-25 | TSSFKFLRTKGSIGHAFTVR | AMP | 0.556 |
| 1 | 7-26 | SSFKFLRTKGSIGHAFTVRI | AMP | 0.705 |
| 1 | 8-27 | SFKFLRTKGSIGHAFTVRIR | AMP | 0.764 |
| 1 | 9-28 | FKFLRTKGSIGHAFTVRIRT | AMP | 0.894 |
| 1 | 10-29 | KFLRTKGSIGHAFTVRIRTG | AMP | 0.867 |
| 1 | 11-30 | FLRTKGSIGHAFTVRIRTGN | AMP | 0.826 |
| 1 | 12-31 | LRTKGSIGHAFTVRIRTGNQ | AMP | 0.551 |
| 1 | 36-55 | FYPFVPHEISVLVELILGHL | AMP | 0.509 |
| 1 | 39-58 | FVPHEISVLVELILGHLRYL | AMP | 0.577 |
| 1 | 40-59 | VPHEISVLVELILGHLRYLL | AMP | 0.588 |
| 1 | 44-63 | ISVLVELILGHLRYLLTDVP | AMP | 0.637 |
| 1 | 46-65 | VLVELILGHLRYLLTDVPPQ | AMP | 0.523 |
| 1 | 72-91 | NVFRPDRPAEAGLGSKKRGG | AMP | 0.508 |
| 1 | 73-92 | VFRPDRPAEAGLGSKKRGGA | AMP | 0.566 |
| 1 | 80-99 | AEAGLGSKKRGGAPPPIHGI | AMP | 0.529 |
| 1 | 81-100 | EAGLGSKKRGGAPPPIHGIS | AMP | 0.545 |
| 1 | 82-101 | AGLGSKKRGGAPPPIHGISK | AMP | 0.858 |
| 1 | 83-102 | GLGSKKRGGAPPPIHGISKI | AMP | 0.949 |

**Results with Artificial Neural Network (ANN) classifier**

| **Seq. ID.** | **Position** | **Sequence** | **Class** |
| --- | --- | --- | --- |
| 1 | 2-21 | NFSDTSSFKFLRTKGSIGHA | AMP |
| 1 | 3-22 | FSDTSSFKFLRTKGSIGHAF | AMP |
| 1 | 6-25 | TSSFKFLRTKGSIGHAFTVR | AMP |
| 1 | 7-26 | SSFKFLRTKGSIGHAFTVRI | AMP |
| 1 | 8-27 | SFKFLRTKGSIGHAFTVRIR | AMP |
| 1 | 9-28 | FKFLRTKGSIGHAFTVRIRT | AMP |
| 1 | 10-29 | KFLRTKGSIGHAFTVRIRTG | AMP |
| 1 | 11-30 | FLRTKGSIGHAFTVRIRTGN | AMP |
| 1 | 71-90 | DNVFRPDRPAEAGLGSKKRG | AMP |
| 1 | 72-91 | NVFRPDRPAEAGLGSKKRGG | AMP |
| 1 | 73-92 | VFRPDRPAEAGLGSKKRGGA | AMP |
| 1 | 74-93 | FRPDRPAEAGLGSKKRGGAP | AMP |
| 1 | 75-94 | RPDRPAEAGLGSKKRGGAPP | AMP |
| 1 | 77-96 | DRPAEAGLGSKKRGGAPPPI | AMP |
| 1 | 78-97 | RPAEAGLGSKKRGGAPPPIH | AMP |
| 1 | 79-98 | PAEAGLGSKKRGGAPPPIHG | AMP |
| 1 | 80-99 | AEAGLGSKKRGGAPPPIHGI | AMP |
| 1 | 81-100 | EAGLGSKKRGGAPPPIHGIS | AMP |
| 1 | 82-101 | AGLGSKKRGGAPPPIHGISK | AMP |
| 1 | 83-102 | GLGSKKRGGAPPPIHGISKI | AMP |

**Results with Discriminant Analysis classifier**

| **Seq. ID.** | **Position** | **Sequence** | **Class** | **AMP Probability** |
| --- | --- | --- | --- | --- |
| 1 | 2-21 | NFSDTSSFKFLRTKGSIGHA | AMP | 0.629 |
| 1 | 3-22 | FSDTSSFKFLRTKGSIGHAF | AMP | 0.615 |
| 1 | 6-25 | TSSFKFLRTKGSIGHAFTVR | AMP | 0.846 |
| 1 | 7-26 | SSFKFLRTKGSIGHAFTVRI | AMP | 0.931 |
| 1 | 8-27 | SFKFLRTKGSIGHAFTVRIR | AMP | 0.960 |
| 1 | 9-28 | FKFLRTKGSIGHAFTVRIRT | AMP | 0.937 |
| 1 | 10-29 | KFLRTKGSIGHAFTVRIRTG | AMP | 0.962 |
| 1 | 11-30 | FLRTKGSIGHAFTVRIRTGN | AMP | 0.922 |
| 1 | 12-31 | LRTKGSIGHAFTVRIRTGNQ | AMP | 0.521 |
| 1 | 39-58 | FVPHEISVLVELILGHLRYL | AMP | 0.515 |
| 1 | 72-91 | NVFRPDRPAEAGLGSKKRGG | AMP | 0.729 |
| 1 | 73-92 | VFRPDRPAEAGLGSKKRGGA | AMP | 0.895 |
| 1 | 74-93 | FRPDRPAEAGLGSKKRGGAP | AMP | 0.514 |
| 1 | 80-99 | AEAGLGSKKRGGAPPPIHGI | AMP | 0.711 |
| 1 | 81-100 | EAGLGSKKRGGAPPPIHGIS | AMP | 0.548 |
| 1 | 82-101 | AGLGSKKRGGAPPPIHGISK | AMP | 0.965 |
| 1 | 83-102 | GLGSKKRGGAPPPIHGISKI | AMP | 0.989 |

| **Predicted Antimicrobial region with translated peptide sequence of Ds-DbRCaG-11-Rga15p**  **Results with Support Vector Machine (SVM) classifier**  NAMP  **Results with Random Forest Classifier**  NAMP  **Results with Artificial Neural Network (ANN) classifier**   \| **Seq. ID.** \| **Position** \| **Sequence** \| **Class** \| \| --- \| --- \| --- \| --- \| \| 1 \| 6-25 \| DNVFRGIDRPTPVMGTKRRA \| AMP \| \| 1 \| 7-26 \| NVFRGIDRPTPVMGTKRRAV \| AMP \| \| 1 \| 10-29 \| RGIDRPTPVMGTKRRAVPRL \| AMP \| \| 1 \| 11-30 \| GIDRPTPVMGTKRRAVPRLR \| AMP \|   **Results with Discriminant Analysis classifier**   \| **Seq. ID.** \| **Position** \| **Sequence** \| **Class** \| **AMP Probability** \| \| --- \| --- \| --- \| --- \| --- \| \| 1 \| 8-27 \| VFRGIDRPTPVMGTKRRAVP \| AMP \| 0.506 \| \| 1 \| 9-28 \| FRGIDRPTPVMGTKRRAVPR \| AMP \| 0.546 \| \| 1 \| 12-31 \| IDRPTPVMGTKRRAVPRLRF \| AMP \| 0.516 \|   Top of Form  Bottom of Form |
| --- | --- | --- | --- | --- | --- | --- | --- | --- | --- | --- | --- | --- | --- | --- | --- | --- | --- | --- | --- | --- | --- | --- | --- | --- | --- | --- | --- | --- | --- | --- | --- | --- | --- | --- | --- | --- | --- | --- | --- | --- |

Top of Form

Bottom of Form
